# A faster way to make GFP-based biosensors: Two new transposons for creating multicolored libraries of fluorescent fusion proteins

**DOI:** 10.1186/1472-6750-4-17

**Published:** 2004-08-18

**Authors:** Douglas L Sheridan, Thomas E Hughes

**Affiliations:** 1 Interdepartmental Neuroscience Program, Yale University, New Haven, Connecticut USA 06520; 2 Department of Cell Biology and Neuroscience, Montana State University, Bozeman, Montana USA 59717

## Abstract

**Background:**

There are now several ways to generate fluorescent fusion proteins by randomly inserting DNA encoding the Green Fluorescent Protein (GFP) into another protein's coding sequence. These approaches can be used to map regions in a protein that are permissive for GFP insertion or to create novel biosensors. While remarkably useful, the current insertional strategies have two major limitations: (1) they only produce one kind, or color, of fluorescent fusion protein and (2) one half of all *GFP *insertions within the target coding sequence are in the wrong orientation.

**Results:**

We have overcome these limitations by incorporating two different fluorescent proteins coding sequences in a single transposon, either in tandem or antiparallel. Our initial tests targeted two mammalian integral membrane proteins: the voltage sensitive motor, Prestin, and an ER ligand gated Ca^2+ ^channel (IP_3_R).

**Conclusions:**

These new designs increase the efficiency of random fusion protein generation in one of two ways: (1) by creating two different fusion proteins from each insertion or (2) by being independent of orientation.

## Background

Biosensors based on GFP-fusion proteins are powerful tools for observing real-time events within living cells. Insertion of GFP within another protein has produced biosensors capable of signaling intracellular events through intrinsic fluorescence changes [1,2], fluorescence resonance energy transfer (FRET) [3,4], and changes in sub-cellular localization [5]. The difficult task of finding the right insertion site to produce a biosensor can be accelerated by screening libraries of random GFP insertions [6-8]. The insertional strategies described to date, however, are limited in two ways. First, each insertion produces only one kind, or color, of fluorescent fusion protein. Creating the multicolored libraries necessary for co-expression or FRET analyses requires either separate rounds of insertion and screening for each fluorescent protein or additional subcloning to exchange fluorophores. Second, the efficiency of any random approach is limited to a maximum of 1:6 because a fusion protein can only be produced if the *GFP *coding sequence lands in the correct orientation and reading frame with respect to the target coding sequence. We reasoned that it might be possible to overcome these limitations by placing two different fluorescent protein coding sequences in a single transposon, either in tandem or antiparallel. Here we present the results of our initial tests with these designs.

## Results and Discussion

**Figure 1:**
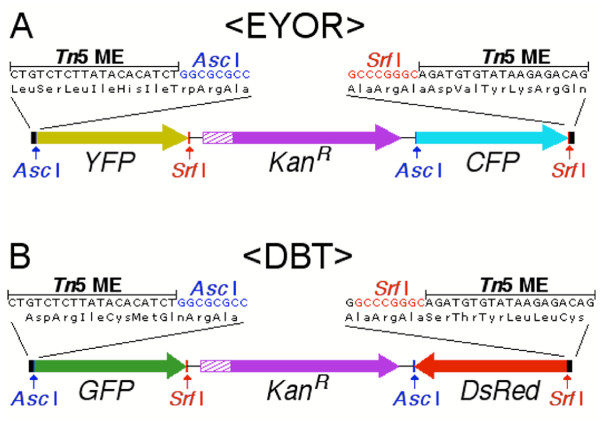
**Two new transposons for generating multicolored GFP fusion protein libraries. **In-frame insertions of the Either-Or transposon, <EYOR> (A), within another coding sequence initially create truncated C-terminal YFP fusions. Alternate restriction digestion with *Asc *I or *Srf *I removes one of the fluorescent proteins and the *Kan*^*R*^. Subsequent re-ligation produces identical full-length YFP and CFP fusion proteins. The fluorescent protein is flanked by 9 amino acid linkers encoded by the Tn*5 *MEs and restriction sites. The Double Barrel transposon, <DBT> (B), encodes green and red fluorescent proteins (GFP and DsRed) antiparallel to one another. Therefore, <DBT> insertions with another coding sequence have a 1:3 chance of being in-frame regardless of orientation. The *GFP *coding sequence uses the same reading frame through the Tn*5 *MEs as *YFP *in <EYOR>. *DsRed *however, has been shifted by 1 bp to use a different reading frame through the Tn*5 *MEs, doubling the number of usable insertion sites within a target coding sequence **(Note that the reading frame shown in <DBT> is that used for *DsRed*, translated from the lower DNA strand, and is read from right to left)**.

The mosaic ends (MEs) that define the hyperactive Tn*5 *transposon [9] have two possible open reading frames (ORFs) through them. We used one of these reading frames to construct the Either-Or transposon (<EYOR>, Figure 1A). <EYOR> carries the sequence encoding the yellow fluorescent protein (YFP) at its 5' end, flanked by two 8 bp restriction sites (*Asc *I – 5' and *Srf *I – 3'). An identical cassette encoding cyan fluorescent protein (CFP) flanked with *Asc *I and *Srf *I sites, is positioned in the same orientation at the 3' end of the transposon. Tn*5 *transposition, *in vitro*, is only ~1% efficient [10], so the kanamycin resistance gene (*Kan*^*R*^) was incorporated between the two fluorescent protein cassettes. Since the *YFP *sequence has no start codon, it should only be translated if it inserts within another protein coding sequence in the correct orientation and reading frame. Plasmids with transposon insertions that are in-frame with respect to the target coding sequence can be rapidly identified by screening for YFP fluorescence in transiently transfected mammalian cells. Each of these clones produces a truncated fusion protein with YFP at the C-terminus. A stop codon is positioned downstream of the *Srf *I site to prevent translation beyond the YFP coding sequence. These truncated fusion proteins may provide additional information about which parts of the primary sequence contain trafficking signals. Full-length YFP and CFP fusion proteins are then generated in parallel from each clone by digestion with *Srf *I or *Asc *I and re-ligation. By producing identical full-length YFP and CFP fusion proteins from each in-frame insertion <EYOR> should double the efficiency with which multicolored fusion protein libraries can be generated.

**Figure 2:**
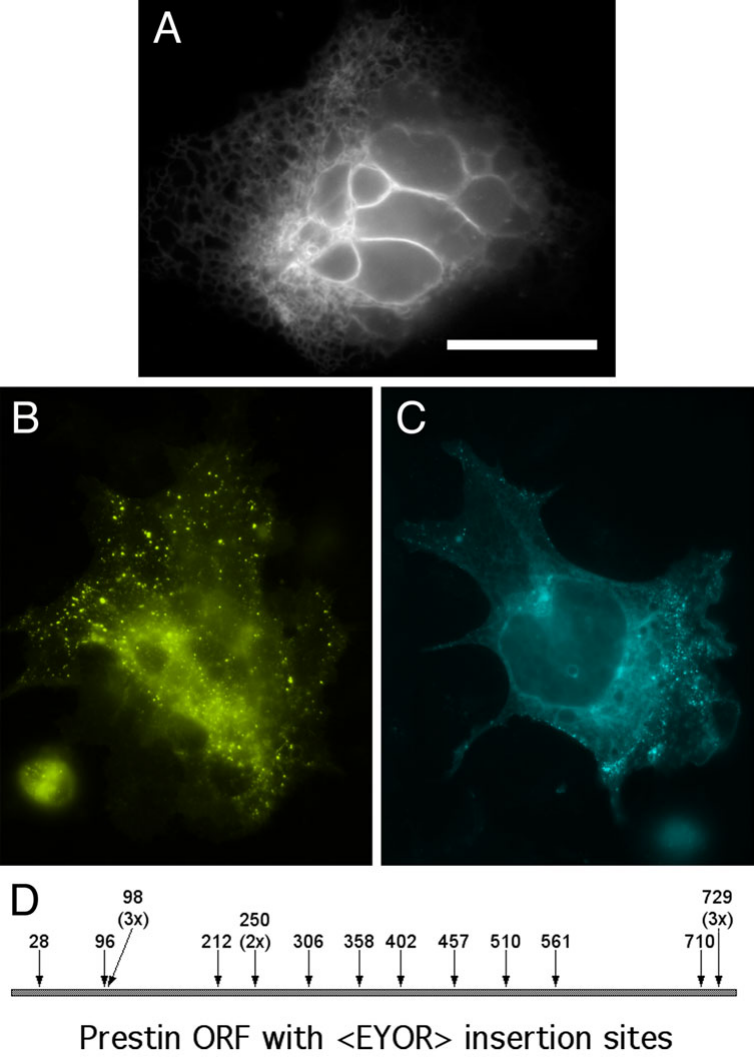
**In-frame <EYOR> transposition events in the voltage sensitive integral membrane motor protein, Prestin. **Visual screening of transiently transfected HEK-293 cells revealed truncated Prestin-YFP fusion proteins generated by in-frame <EYOR> transpositions (A) (scale bar = 20 μm). Alternate digestion and re-ligation of an in-frame clone with either *Srf *I or *Asc *I produces identical full-length YFP- (B) and CFP-fusion proteins (C). In-frame <EYOR> insertions in Prestin are identified by the first of 3 target amino acids duplicated during transposition (D). Redundant insertions are indicated by the number of recovered clones (e.g. 3x).

To test the <EYOR> transposon we targeted Prestin, an integral membrane protein expressed in outer hair cells of the cochlea and believed to be the motor responsible for their rapid changes in length in response to fluctuations in membrane voltage [11]. The 2.2 kb cDNA encoding Prestin was expressed in a 4.8 kb Ampicillin resistant (*Amp*^*R*^) CMV expression plasmid, pBNJ12.5. Transposon insertions that disrupt the plasmid origin or *Amp*^*R *^(together ~1.5 kb) are not recovered [8], so the predicted number of in-frame insertions in Prestin, is ~7%. After transposition, plasmids conferring Amp^R ^and Kan^R ^were isolated by standard mini-prep procedures and transiently expressed in HEK-293 cells in a 96-well format. In a random sample of 192 transposed clones, 32 produced detectable fluorescence. Eighteen of these (~9%) were clearly localized to intracellular membranes. (Figure 2A). Of the remaining fluorescent proteins 11 appeared to be YFP alone, with evenly distributed fluorescence throughout the entire cell, and 3 were too dim to determine any sub-cellular localization. Sequencing revealed that the 18 proteins targeted to intracellular membranes were truncated Prestin-YFP fusion proteins resulting from in-frame insertions at 12 unique sites (Figure 2D). One of the YFP-like clones was an in-frame insertion in the intracellular N-terminus of Prestin (at amino acids 28–30) upstream of the first predicted transmembrane domain. The remaining 13 fluorescent proteins resulted from <EYOR> insertions outside the Prestin coding sequence, most of them being clustered just downstream of the CMV promoter. We could not identify an in-frame start codon (AUG) in any of these clones. There is however, an in-frame CUG codon at the 5' end of the *Tn*5 ME sequence that may present an alternate translation initiation site in the presence of the strong CMV promoter [12].

To verify that <EYOR> could be used to generate full-length fusions with either YFP or CFP unique in-frame clones were digested in parallel with *Srf *I or *Asc *I and re-ligated. The resulting fusion constructs were transiently expressed in HEK-293 cells and screened for YFP and CFP fluorescence (Figure 2B,2C). All 13 unique insertion sites produced fluorescent full-length fusions with both CFP and YFP and all were localized to intracellular membranes.

The <EYOR> design could be expanded for a wide range of protein tagging applications by replacing the secondary CFP cassette with another open reading frame. With such a transposon, YFP fluorescence would be used as a reporter to rapidly identify random in-frame insertions. Subsequent digestion with *Asc *I and re-ligation could then generate fusion proteins that might otherwise be difficult to screen for such as epitope tags, protease cleavage sites, or even a new N-terminus complete with a secretory signal peptide. Several groups have reported similar strategies based on multi-domain transposons for the random insertion of small peptide tags [reviewed in:[13]]. Like <EYOR>, these transposons utilize a primary reporter domain to identify in-frame insertions. Subsequent excision of the reporter domain (and selectable marker) then restores the full-length target coding sequence with an inserted peptide tag. The <EYOR> design is unique, however, in that its overlapping pairs of *Asc *I and *Srf *I restriction sites, allow the user to create identical full-length fusion proteins from both the reporter domain and the secondary coding sequence.

The second transposon design, the Double-Barrel transposon (<DBT>, Figure 1B), encodes green and red fluorescent proteins (GFP and DsRed) in opposite orientations. This means that any <DBT> insertion within another protein coding sequence has a 1:3 chance of being in-frame regardless of its orientation. Therefore, <DBT> should double the efficiency of random fusion protein generation, by producing equal numbers of GFP and DsRed fusions.

In addition to their antiparallel orientation, the *GFP *and *DsRed *coding sequences in <DBT> each use a different relative reading frame through the Tn*5 *MEs. As in <EYOR>, GFP fusion proteins are created by insertions after the third nucleotide of a target codon. The *DsRed *coding sequence, however, has been shifted by 1 nucleotide relative to the Tn*5 *MEs. Therefore, DsRed fusion proteins are generated by transposon insertions between the second and third nucleotides. Using different reading frames for *GFP *and *DsRed *doubles the total number of insertion sites in the target coding sequence from which fusion proteins could potentially be made. While this does not alter the frequency of in-frame insertions, it does reduce the screening cost of saturating a target clone by increasing the probability of recovering unique in-frame insertions.

**Figure 3:**
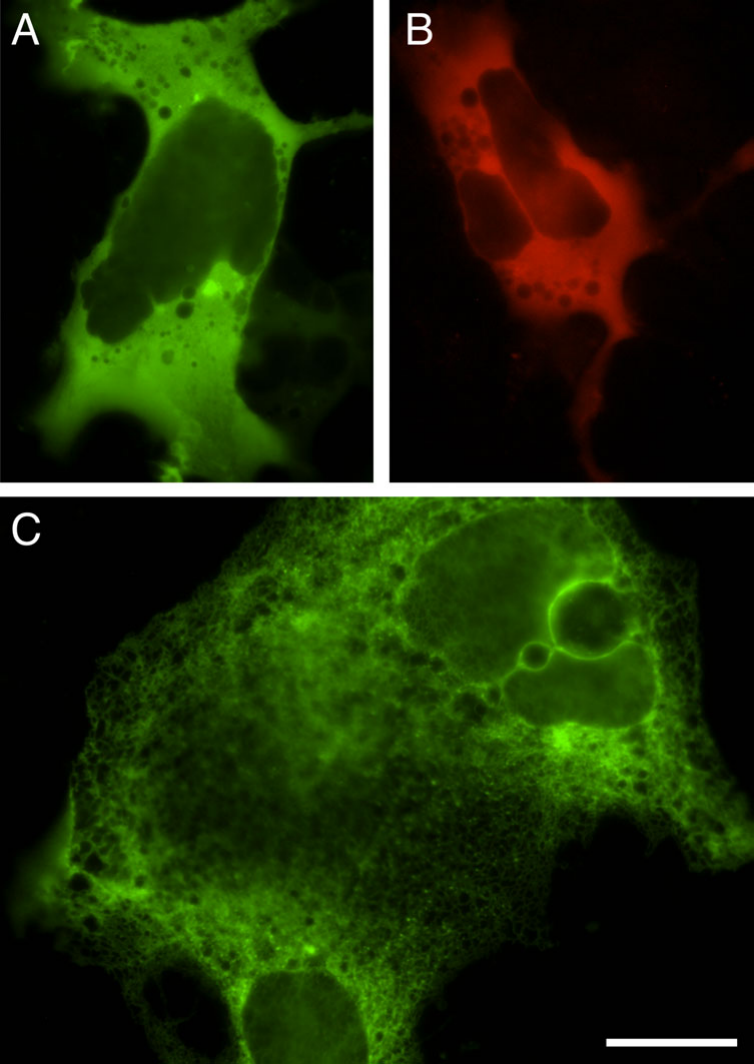
**Transposition of the type 1 IP_3_R with the Double Barrel Transposon (<DBT>). **Transient expression in HEK-293 cells allows identification of in-frame insertions producing truncated GFP- (A) or DsRed- (B) IP_3_R fusion proteins. Digestion and subsequent re-ligation produces a full-length fluorescent IP_3_R fusion protein (C) (Scale bar = 20 μm).

To test the efficiency of fusion protein generation with the <DBT> transposon, we targeted pCMVI-9, a CMV expression plasmid carrying cDNA encoding the type 1 IP_3 _receptor (IP_3_R) [14]. The IP_3_R is a ligand gated Ca^2+ ^channel, composed of 4 homomeric subunits, expressed within the membranes of the endoplasmic reticulum. Each IP_3_R subunit is over 2700 amino acids, and creating a full-length fluorescent IP_3_R fusion protein with such a large cDNA presents a formidable challenge for traditional molecular biological techniques. The high ratio of coding sequence to vector makes it an excellent target for transposition, however, with a predicted frequency of in-frame insertions of ~11% per fluorophore for <DBT>. At 24 hrs post-transfection, visual screening for fluorescence of 288 Amp^R ^+ Kan^R ^clones in HEK-293 cells identified 44 clones that produced green fluorescent proteins. Of these, 35 displayed a uniform cytoplasmic distribution and exclusion from the nucleus (Figure 3A), 3 showed fluorescence throughout the entire cell and 6 were too dim to determine any sub-cellular localization. Screening at several time points between 2 and 4 days post-transfection identified 7 clones producing red fluorescent proteins, 2 of which were clearly excluded from the nucleus (Figure 3B). The remaining 5 red proteins were too dim to determine any sub-cellular localization. Sequencing out of the transposon confirmed that 41 of the clones encoding green proteins (~14%) and 2 clones encoding red proteins represented in-frame insertions. Consistent with the results of the Prestin transposition, all of the proteins with sub-cellular localization different from that seen with GFP alone were the product of in-frame insertions. After digestion and religation, only 20 of the full-length fusion proteins retained detectable levels of fluorescence (18 green, 2 red). These full-length proteins displayed a dramatic shift in their distribution, with clear ER localization (Figure 3C).

We chose *GFP *and *DsRed *to build the <DBT> transposon because their coding sequences are so dissimilar. Our concern was that if we chose two similar sequences, *CFP *and *YFP *for example, the antiparallel orientation of these coding sequences could produce extensive mRNA hybridization and secondary structure that would inhibit protein translation. It appears however, that DsRed is not well suited for insertion within other proteins. Indeed, DsRed has not been reported as a fusion protein in the middle of another protein, and even N- and C-terminal fusions with DsRed can be problematic [15], perhaps due to its being an obligate multimer [16]. Despite the low yield of DsRed fusions, these results demonstrate that <DBT> can be used to simultaneously generate full-length fusion proteins in two different reading frames. As novel fluorescent proteins are isolated from new species, or old ones are altered, this type of bi-directional transposon could potentially double the output of the screening process.

## Conclusions

The transposons described here should greatly accelerate the creation of multicolored libraries of fluorescent fusion proteins. By creating identical full-length YFP and CFP fusion proteins from each in-frame insertion, the <EYOR> transposon not only facilitates the generation potential FRET pairs, it enables the direct comparison of different fluorophores in otherwise identical fusion proteins. The <DBT> design, on the other hand, has the capacity to double both the throughput of fusion protein generation by virtue of its bi-directionality as well as the total output of novel fusion proteins through the simultaneous use of multiple reading frames. Ultimately, the ability to generate large numbers of novel fusions proteins in days rather than months, should shift the limiting rate at which novel fluorescent protein biosensors are identified to functional screening rather than protein design and construction.

## Methods

### Plasmids

PCR and standard subcloning procedures were used to create the plasmids encoding <EYOR> (pBNJ55b.1), <DBT> (pBNJ38.5) and Prestin (pBNJ12.5) (full sequences in supplementary material). The fluorescent protein coding sequences used were Venus (YFP) [17], ECFP-N164H (CFP), EGFP (GFP) and DsRed2 (Clontech). The *Kan*^*R *^gene was obtained from pUniV5-His-TOPO (Invitrogen). The construction of the IP3 receptor expression plasmid, pCMVI-9, was previously described [14].

### Tn5 transposition and plasmid isolation

Transposons were amplified from their host plasmids via PCR with a single primer complementary to the 19 bp Tn*5 *ME (5'-CTGTCTCTTATACACATCT-3') and purified as previously described [8]. Purified transposon and target concentrations were each quantified against an independent DNA standard using a DynaQuant 200 fluorimeter. The transposition reaction was performed according to manufacturer's recommendations (Epicentre Technologies, Madison, WI) with 200 ng of target DNA and a molar equivalent of purified transposon. Electrocompetent XL-10 Gold *E. coli *(Stratagene, La Jolla, CA) were transformed with 0.5 μL of the transposition reaction and plated on LB agar with Ampicillin (100 μg/mL) and Kanamycin (50 μg/mL). Parallel plating of the transformation on LB agar with Ampicillin alone was used to establish the transposition efficiency.

Transposed plasmids were isolated in a 96-well format from 1.25 mL LB cultures with Eppendorf PerfectPREP-96 Vac Direct Bind miniprep kits on a PerkinElmer MultiPROBE II HT liquid handling robot and eluted in 70 μL of ddH_2_O.

### Visual screening

HEK-293 cells (American Type Culture Collection CRL-1573) were plated 24 hr prior to transfection, in 96-well glass bottom tissue culture plates (NalgeNUNC) at 6 × 10^4 ^cells in 100 μL of MEM-E with 10% fetal bovine serum. Transfections were performed with ~300 ng of plasmid DNA and 0.3 μL of Lipofectamine 2000™ (Gibco BRL) in a total volume of 50 μL of Opti-MEMI (Gibco BRL) per well. The cells were screened for fluorescence 24 hr after transfection with a 20× objective on a Zeiss inverted microscope with excitation and emission filter sets optimized for CFP/YFP or GFP/DsRed imaging (Omega, Brattleboro, VT).

### Sequencing and generation of full-length fusion proteins

Exact insertion sites were identified for all fluorescent transposed clones by sequencing 5' out of the transposon with a primer complimentary to the <EYOR>*YFP *coding region (5'-CTGCAGGCCGTAGCC-3') or <DBT>*GFP *coding region (5'-TGGCCGTTTACGTCGCCGTCCA-3'). To generate full-length fusion proteins, plasmids with unique in-frame insertions were digested and re-ligated. After restriction digestion (100 ng of plasmid DNA and 0.5 U of *Asc *I or *Srf *I in 10 μL total volume), 1 μL of the digest reaction (~20 ng DNA) was re-ligated with Fast-Link™ ligase (Epicentre Technologies) for 15 min at room temperature (0.5 mM ATP, 1X Fast-Link™ buffer, 1 U ligase, 7.5 μL total volume). After heat inactivation (70°C for 15 min.), XL-10 Gold *E. coli *were transformed with 0.5 μL of the ligation reaction and plated on LB agar with Ampicillin. The following day, colonies were co-inoculated in LB with Ampicillin and Ampicillin + Kanamycin to verify loss of the *Kan*^*R *^prior to plasmid isolation.

## Authors' contributions

D.S. conceived of the transposon designs and carried out their construction, performed the transposition and screening for fluorescent fusion proteins, and drafted the manuscript. T.H. participated in the study design, coordination, and analysis. All authors have read and approved the final manuscript.

## Supplementary Material

Additional File 1 **Supplementary Material-hughes **This is a PDF file containing both plasmid maps and full sequence data for the plasmids used in this study. Click here for file
