# Supplementary material for: A faster way to make GFP-based biosensors: Two new transposons for creating multicolored libraries of fluorescent fusion proteins
Source: BMC Biotechnol. 2004 Aug 18;4:17. doi: 10.1186/1472-6750-4-17 (PMC514711; doi:10.1186/1472-6750-4-17)
Supplement: Supplementary file 1 [file 1472-6750-4-17-S1.doc]

pBNJ55b.1

<EYOR> in pcDNA1/Amp (7376 bp)

<EYOR>	3196-640
Tn*5* ME	3196-3178
Venus ORF	3196-2447
Kan promoter	2387-2248
Kan resistance ORF	2249-1455
ECFP ORF	1386-640
Tn*5* ME	658-640
Amp promoter	6296-6333
Amp resistance	6368-7228
pMB1 ori	4993-5575

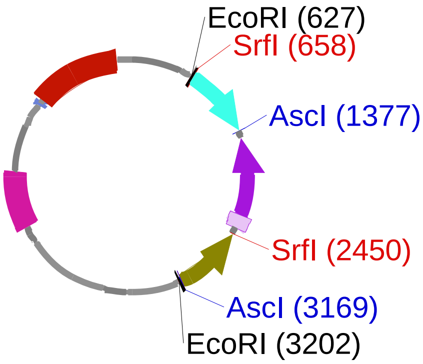

GGCGTAATCTGCTGCTTGCAAACAAAAAAACCACCGCTACCAGCGGTGGTTTGTTTGCCGGATCAAGAGCTACCAACTCTTTTTCCGAAGGTAACTGGCT
TCAGCAGAGCGCAGATACCAAATACTGTCCTTCTAGTGTAGCCGTAGTTAGGCCACCACTTCAAGAACTCTGTAGCACCGCCTACATACCTCGCTCTGCT
AATCCTGTTACCAGTGGCTGCTGCCAGTGGCGATAAGTCGTGTCTTACCGGGTTGGACTCAAGACGATAGTTACCGGATAAGGCGCAGCGGTCGGGCTGA
ACGGGGGGTTCGTGCACACAGCCCAGCTTGGAGCGAACGACCTACACCGAACTGAGATACCTACAGCGTGAGCATTGAGAAAGCGCCACGCTTCCCGAAG
GGAGAAAGGCGGACAGGTATCCGGTAAGCGGCAGGGTCGGAACAGGAGAGCGCACGAGGGAGCTTCCAGGGGGAAACGCCTGGTATCTTTATAGTCCTGT
CGGGTTTCGCCACCTCTGACTTGAGCGTCGATTTTTGTGATGCTCGTCAGGGGGGCGGAGCCTATGGAAAAACGCCAGCAACGCAAGCTAGCTTCTAGCT
AGAAATTGTAAACGTTAATATTTTGTTAAAATTCGCGTTAAATTTTTGTTAAATCAGCTCATTTTTTAACCAATAGGCCGAAATCGGCAAAATCCCTTAT
AAATCAAAAGAATAGCCCGAGATAGGGTTGAGTGTTGTTCCAGTTTGGAACAAGAGTCCACTATTAAAGAACGTGGACTCCAACGTCAAAGGGCGAAAAA
CCGTCTATCAGGGCGATGGCCGCCCACTACGTGAACCATCACCCAAATCAAGTTTTTTGGGGTCGAGGTGCCGTAAAGCACTAAATCGGAACCCTAAAGG
GAGCCCCCGATTTAGAGCTTGACGGGGAAAGCCGGCGAACGTGGCGAGAAAGGAAGGGAAGAAAGCGAAAGGAGCGGGCGCTAGGGCGCTGGCAAGTGTA
GCGGTCACGCTGCGCGTAACCACCACACCCGCCGCGCTTAATGCGCCGCTACAGGGCGCGTACTATGGTTGCTTTGACGAGACCGTATAACGTGCTTTCC
TCGTTGGAATCAGAGCGGGAGCTAAACAGGAGGCCGATTAAAGGGATTTTAGACAGGAACGGTACGCCAGCTGGATTACCAAAGGCCTCGTGATACGCC
TATTTTTATAGGTTAATGTCATGATAATAATGGTTTCTTAGACGTCAGGTGGCACTTTTCGGGGAAATGTGCGCGGAACCCCTATTTGTTTATTTTTCTA
AATACATTCAAATATGTATCCGCTCATGAGACAATAACCCTGATAAATGCTTCAATAATATTGAAAAAGGAAGAGTATGAGTATTCAACATTTCCGTGTC
GCCCTTATTCCCTTTTTTGCGGCATTTTGCCTTCCTGTTTTTGCTCACCCAGAAACGCTGGTGAAAGTAAAAGATGCTGAAGATCAGTTGGGTGCACGAG
TGGGTTACATCGAACTGGATCTCAACAGCGGTAAGATCCTTGAGAGTTTTCGCCCCGAAGAACGTTTTCCAATGATGAGCACTTTTAAAGTTCTGCTATG
TGGCGCGGTATTATCCCGTGTTGACGCCGGGCAAGAGCAACTCGGTCGCCGCATACACTATTCTCAGAATGACTTGGTTGAGTACTCACCAGTCACAGAA
AAGCATCTTACGGATGGCATGACAGTAAGAGAATTATGCAGTGCTGCCATAACCATGAGTGATAACACTGCGGCCAACTTACTTCTGACAACGATCGGAG
GACCGAAGGAGCTAACCGCTTTTTTGCACAACATGGGGGATCATGTAACTCGCCTTGATCGTTGGGAACCGGAGCTGAATGAAGCCATACCAAACGACGA
GCGTGACACCACGATGCCTGCAGCAATGGCAACAACGTTGCGCAAACTATTAACTGGCGAACTACTTACTCTAGCTTCCCGGCAACAATTAATAGACTGG
ATGGAGGCGGATAAAGTTGCAGGACCACTTCTGCGCTCGGCCCTTCCGGCTGGCTGGTTTATTGCTGATAAATCTGGAGCCGGTGAGCGTGGGTCTCGCG
GTATCATTGCAGCACTGGGGCCAGATGGTAAGCCCTCCCGTATCGTAGTTATCTACACGACGGGGAGTCAGGCAACTATGGATGAACGAAATAGACAGAT
CGCTGAGATAGGTGCCTCACTGATTAAGCATTGGTAACTGTCAGACCAAGTTTACTCATATATACTTTAGATTGATTTAAAACTTCATTTTTAATTTCTC
TAGCGCGTTGACATTGATTATTGACTAGTTATTAATAGTAATCAATTACGGGGTCATTAGTTCATAGCCCATATATGGAGTTCCGCGTTACATAACTTAC
GGTAAATGGCCCGCCTGGCTGACCGCCCAACGACCCCCGCCCATTGACGTCAATAATGACGTATGTTCCCATAGTAACGCCAATAGGGACTTTCCATTGA
CGTCAATGGGTGGACTATTTACGGTAAACTGCCCACTTGGCAGTACATCAAGTGTATCATATGCCAAGTACGCCCCCTATTGACGTCAATGACGGTAAAT
GGCCCGCCTGGCATTATGCCCAGTACATGACCTTATGGGACTTTCCTACTTGGCAGTACATCTACGTATTAGTCATCGCTATTACCATGGTGATGCGGTT
TTGGCAGTACATCAATGGGCGTGGATAGCGGTTTGACTCACGGGGATTTCCAAGTCTCCACCCCATTGACGTCAATGGGAGTTTGTTTTGGCACCAAAAT
CAACGGGACTTTCCAAAATGTCGTAACAACTCCGCCCCATTGACGCAAATGGGCGGTAGGCGTGTACGGTGGGAGGTCTATATAAGCAGAGCTCTCTGGC
TAACTAGAGAACCCACTGCTTACTGGCTTATCGAAATTAATACGACTCACTATAGGGAGACCCAAGCTTGGTACCGAGCTCGGATCCACTAGTAACGGCC
GCCAGTGTGCTGGAATTCGCCCTTCTGTCTCTTATACACATCTGCCCGGGCCTTGTACAGCTCGTCCATGCCGAGAGTGATCCCGGCGGCGGTCACGAAC
TCCAGCAGGACCATGTGATCGCGCTTCTCGTTGGGGTCTTTGCTCAGGGCGGACTGGGTGCTCAGGTAGTGGTTGTCGGGCAGCAGCACGGGGCCGTCGC
CGATGGGGGTGTTCTGCTGGTAGTGGTCGGCGAGCTGCACGCTGCCGTCCTCGATGTTGTGGCGGATCTTGAAGTGGGCCTTGATGCCGTTCTTCTGCTT
GTCGGCGGTGATATAGACGTTGTGGCTGATGTAGTTGTACTCCAGCTTGTGCCCCAGGATGTTGCCGTCCTCCTTGAAGTCGATGCCCTTCAGCTCGATG
CGGTTCACCAGGGTGTCGCCCTCGAACTTCACCTCGGCGCGGGTCTTGTAGTTGCCGTCGTCCTTGAAGAAGATGGTGCGCTCCTGGACGTAGCCTTCGG
GCATGGCGGACTTGAAGAAGTCGTGCTGCTTCATGTGGTCGGGGTAGCGGCTGAAGCACTGCACGCCCCAGGTCAGGGTGGTCACGAGGGTGGGCCAGGG
CACGGGCAGCTTGCCGGTGGTGCAGATGAACTTCAGGGTCAGCTTGCCGTAGGTGGCATCGCCCTCGCCCTCGCCGGACACGCTGAACTTGTGGCCGTTT
ACGTCGCCGTCCAGCTCGACCAGGATGGGCACCACCCCGGTGAACAGCTCCTCGCCCTTGCTGGCGCGCCTGTAATCGAAATCTCGTGATGGCAGGTTGG
GCGTCGCTTGGTCGGTCATTTCGAACCCCAGAGTCCCGCTCAGAAGAACTCGTCAAGAAGGCGATAGAAGGCGATGCGCTGCGAATCGGGAGCGGCGATA
CCGTAAAGCACGAGGAAGCGGTCAGCCCATTCGCCGCCAAGCTCTTCAGCAATATCACGGGTAGCCAACGCTATGTCCTGATAGCGGTCCGCCACACCCA
GCCGGCCACAGTCGATGAATCCAGAAAAGCGGCCATTTTCCACCATGATATTCGGCAAGCAGGCATCGCCATGTGTCACGACGAGATCCTCGCCGTCGGG
CATGCGCGCCTTGAGCCTGGCGAACAGTTCGGCTGGCGCGAGCCCCTGATGCTCTTCGTCCAGATCATCCTGATCGACAAGACCGGCTTCCATCCGAGTA
CGTGCTCGCTCGATGCGATGTTTCGCTTGGTGGTCGAATGGGCAGGTAGCCGGATCAAGCGTATGCAGCCGCCGCATTGCATCAGCCATGATGGATACTT
TCTCGGCAGGAGCAAGGTGAGATGACAGGAGATCCTGCCCCGGCACTTCGCCCAATAGCAGCCAGTCCCTTCCCGCTTCAGTGACAACGTCGAGCACAGC
TGCGCAAGGAACGCCCGTCGTGGCCAGCCACGATAGCCGCGCTGCCTCGTCCTGCAGTTCATTCAGGGCACCGGACAGGTCGGTCTTGACAAAAAGAACC
GGGCGCCCCTGCGCTGACAGCCGGAACACGGCGGCATCAGAGCAGCCGATTGTCTGTTGTGCCCAGTCATAGCCGAATAGCCTCTCCACCCAAGCGGCCG
GAGAACCTGCGTGCAATCCATCTTGTTCAATCATGCGAAACGATCCTCATCCTGTCTCTTGATCAGATCTTGATCCCCTGCGCCATCAGATCCTTGGCGG
CAAGAAAGCCATCCAGTTTACTTTGCAGGGCTTCCCAACCTTACCAGAGGGCGCCCCAGCTGGCAATTCCGGTTCGCTTGCTGTCCATAAAACCGCCCAG
TCTAGCTATCGCCATGTAAGCCCACTGCAAGCTACGCCCGGGCCTTGTACAGCTCGTCCATGCCGAGAGTGATCCCGGCGGCGGTCACGAACTCCAGCAG
GACCATGTGATCGCGCTTCTCGTTGGGGTCTTTGCTCAGGGCGGACTGGTAGCTCAGGTAGTGGTTGTCGGGCAGCAGCACGGGGCCGTCGCCGATGGGG
GTGTTCTGCTGGTAGTGGTCGGCGAGCTGCACGCCGCCGTCCTCGATGTTGTGGCGGATCTTGAAGTTGGCCTTGATGCCGTTCTTCTGCTTGTCGGCGG
TGATATAGACGTTGTGGCTGTTGTAGTTGTACTCCAGCTTGTGCCCCAGGATGTTGCCGTCCTCCTTGAAGTCGATGCCCTTCAGCTCGATGCGGTTCAC
CAGGGTGTCGCCCTCGAACTTCACCTCGGCGCGGGTCTTGTAGTTGCCGTCGTCCTTGAAGAAGATGGTGCGCTCCTGGACGTAGCCTTCGGGCATGGCG
GACTTGAAGAAGTCGTGCTGCTTCATGTGGTCGGGGTAGCGGGCGAAGCACTGCAGGCCGTAGCCCAGGGTGGTCACGAGGGTGGGCCAGGGCACGGGCA
GCTTGCCGGTGGTGCAGATCAGCTTCAGGGTCAGCTTGCCGTAGGTGGCATCGCCCTCGCCCTCGCCGGACACGCTGAACTTGTGGCCGTTTACGTCGCC
GTCCAGCTCGACCAGGATGGGCACCACCCCGGTGAACAGCTCCTCGCCCTTGCTGGCGCGCCAGATGTGTATAAGAGACAGAAGGGCGAATTCTGCAGAT
ATCCATCACACTGGCGGCCGCTCGAGCATGCATCTAGAGGGCCCTATTCTATAGTGTCACCTAAATGCTAGAGGATCTTTGTGAAGGAACCTTACTTCTG
TGGTGTGACATAATTGGACAAACTACCTACAGAGATTTAAAGCTCTAAGGTAAATATAAAATTTTTAAGTGTATAATGTGTTAAACTACTGATTCTAATT
GTTTGTGTATTTTAGATTCCAACCTATGGAACTGATGAATGGGAGCAGTGGTGGAATGCCTTTAATGAGGAAAACCTGTTTTGCTCAGAAGAAATGCCAT
CTAGTGATGATGAGGCTACTGCTGACTCTCAACATTCTACTCCTCCAAAAAAGAAGAGAAAGGTAGAAGACCCCAAGGACTTTCCTTCAGAATTGCTAAG
TTTTTTGAGTCATGCTGTGTTTAGTAATAGAACTCTTGCTTGCTTTGCTATTTACACCACAAAGGAAAAAGCTGCACTGCTATACAAGAAAATTATGGAA
AAATATTTGATGTATAGTGCCTTGACTAGAGATCATAATCAGCCATACCACATTTGTAGAGGTTTTACTTGCTTTAAAAAACCTCCCACACCTCCCCCTG
AACCTGAAACATAAAATGAATGCAATTGTTGTTGTTAACTTGTTTATTGCAGCTTATAATGGTTACAAATAAAGCAATAGCATCACAAATTTCACAAATA
AAGCATTTTTTTCACTGCATTCTAGTTGTGGTTTGTCCAAACTCATCAATGTATCTTATCATGTCTGGATCATCCCGCCATGGTATCAACGCCATATTTC
TATTTACAGTAGGGACCTCTTCGTTGTGTAGGTACCGCTGTATTCCTAGGGAAATAGTAGAGGCACCTTGAACTGTCTGCATCAGCCATATAGCCCCCGC
TGTTCGACTTACAAACACAGGCACAGTACTGACAAACCCATACACCTCCTCTGAAATACCCATAGTTGCTAGGGCTGTCTCCGAACTCATTACACCCTCC
AAAGTCAGAGCTGTAATTTCGCCATCAAGGGCAGCGAGGGCTTCTCCAGATAAAATAGCTTCTGCCGAGAGTCCCGTAAGGGTAGACACTTCAGCTAATC
CCTCGATGAGGTCTACTAGAATAGTCAGTGCGGCTCCCATTTTGAAAATTCACTTACTTGATCAGCTTCAGAAGATGGCGGAGGGCCTCCAACACAGTAA
TTTTCCTCCCGACTCTTAAAATAGAAAATGTCAAGTCAGTTAAGCAGGAAGTGGACTAACTGACGCAGCTGGCCGTGCGACATCCTCTTTTAATTAGTTG
CTAGGCAACGCCCTCCAGAGGGCGTGTGGTTTTGCAAGAGGAAGCAAAAGCCTCTCCACCCAGGCCTAGAATGTTTCCACCCAATCATTACTATGACAAC
AGCTGTTTTTTTTAGTATTAAGCAGAGGCCGGGGACCCCTGGGCCCGCTTACTCTGGAGAAAAAGAAGAGAGGCATTGTAGAGGCTTCCAGAGGCAACTT
GTCAAAACAGGACTGCTTCTATTTCTGTCACACTGTCTGGCCCTGTCACAAGGTCCAGCACCTCCATACCCCCTTTAATAAGCAGTTTGGGAACGGGTGC
GGGTCTTACTCCGCCCATCCCGCCCCTAACTCCGCCCAGTTCCGCCCATTCTCCGCCCCATGGCTGACTAATTTTTTTTATTTATGCAGAGGCCGAGGCC
GCCTCGGCCTCTGAGCTATTCCAGAAGTAGTGAGGAGGCTTTTTTGGAGGCCTAGGCTTTTGCAAAAAGCTAATTC

pBNJ38.5

<DBT> in Kanamycin/Zeocin resisant pCRBluntII-TOPO plasmid (6034 bp)

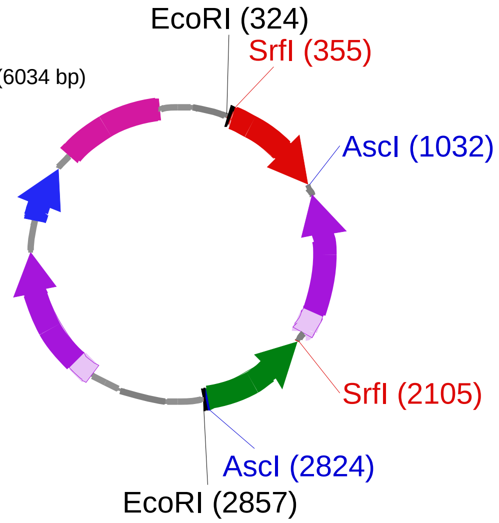

<DBT>	337-2851
Tn*5* ME	337-355
DsRed2 ORF	338-1046
Kan resistance ORF	1904-1110
Kan promoter	2042-1903
EGFP ORF	2851-2102
Tn*5* ME	2851-2833
Kan/Zeo promoter	3614-3751
Kan resistance	3752-4546
Zeo resistance	4753-5127
pMB1 ori	5238-5912

AGCGCCCAATACGCAAACCGCCTCTCCCCGCGCGTTGGCCGATTCATTAATGCAGCTGGCACGACAGGTTTCCCGACTGGAAAGCGGGCAGTGAGCGCAA
CGCAATTAATGTGAGTTAGCTCACTCATTAGGCACCCCAGGCTTTACACTTTATGCTTCCGGCTCGTATGTTGTGTGGAATTGTGAGCGGATAACAATTT
CACACAGGAAACAGCTATGACCATGATTACGCCAAGCTATTTAGGTGACACTATAGAATACTCAAGCTATGCATCAAGCTTGGTACCGAGCTCGGATCCA
CTAGTAACGGCCGCCAGTGTGCTGGAATTCGCCCTTCTGTCTCTTATACACATCTGCCCGGGCCTCCTCCGAGAACGTCATCACCGAGTTCATGCGCTTC
AAGGTGCGCATGGAGGGCACCGTGAACGGCCACGAGTTCGAGATCGAGGGCGAGGGCGAGGGCCGCCCCTACGAGGGCCACAACACCGTGAAGCTGAAGG
TGACCAAGGGCGGCCCCCTGCCCTTCGCCTGGGACATCCTGTCCCCCCAGTTCCAGTACGGCTCCAAGGTGTACGTGAAGCACCCCGCCGACATCCCCGA
CTACAAGAAGCTGTCCTTCCCCGAGGGCTTCAAGTGGGAGCGCGTGATGAACTTCGAGGACGGCGGCGTGGCGACCGTGACCCAGGACTCCTCCCTGCAG
GACGGCTGCTTCATCTACAAGGTGAAGTTCATCGGCGTGAACTTCCCCTCCGACGGCCCCGTGATGCAGAAGAAGACCATGGGCTGGGAGGCCTCCACCG
AGCGCCTGTACCCCCGCGACGGCGTGCTGAAGGGCGAGACCCACAAGGCCCTGAAGCTGAAGGACGGCGGCCACTACCTGGTGGAGTTCAAGTCCATCTA
CATGGCCAAGAAGCCCGTGCAGCTGCCCGGCTACTACTACGTGGACGCCAAGCTGGACATCACCTCCCACAACGAGGACTACACCATCGTGGAGCAGTAC
GAGCGCACCGAGGGCCGCCACCACCTGTTCCTGGCGCGCCTGTAATCGAAATCTCGTGATGGCAGGTTGGGCGTCGCTTGGTCGGTCATTTCGAACCCCA
GAGTCCCGCTCAGAAGAACTCGTCAAGAAGGCGATAGAAGGCGATGCGCTGCGAATCGGGAGCGGCGATACCGTAAAGCACGAGGAAGCGGTCAGCCCAT
TCGCCGCCAAGCTCTTCAGCAATATCACGGGTAGCCAACGCTATGTCCTGATAGCGGTCCGCCACACCCAGCCGGCCACAGTCGATGAATCCAGAAAAGC
GGCCATTTTCCACCATGATATTCGGCAAGCAGGCATCGCCATGTGTCACGACGAGATCCTCGCCGTCGGGCATGCGCGCCTTGAGCCTGGCGAACAGTTC
GGCTGGCGCGAGCCCCTGATGCTCTTCGTCCAGATCATCCTGATCGACAAGACCGGCTTCCATCCGAGTACGTGCTCGCTCGATGCGATGTTTCGCTTGG
TGGTCGAATGGGCAGGTAGCCGGATCAAGCGTATGCAGCCGCCGCATTGCATCAGCCATGATGGATACTTTCTCGGCAGGAGCAAGGTGAGATGACAGGA
GATCCTGCCCCGGCACTTCGCCCAATAGCAGCCAGTCCCTTCCCGCTTCAGTGACAACGTCGAGCACAGCTGCGCAAGGAACGCCCGTCGTGGCCAGCCA
CGATAGCCGCGCTGCCTCGTCCTGCAGTTCATTCAGGGCACCGGACAGGTCGGTCTTGACAAAAAGAACCGGGCGCCCCTGCGCTGACAGCCGGAACACG
GCGGCATCAGAGCAGCCGATTGTCTGTTGTGCCCAGTCATAGCCGAATAGCCTCTCCACCCAAGCGGCCGGAGAACCTGCGTGCAATCCATCTTGTTCAA
TCATGCGAAACGATCCTCATCCTGTCTCTTGATCAGATCTTGATCCCCTGCGCCATCAGATCCTTGGCGGCAAGAAAGCCATCCAGTTTACTTTGCAGGG
CTTCCCAACCTTACCAGAGGGCGCCCCAGCTGGCAATTCCGGTTCGCTTGCTGTCCATAAAACCGCCCAGTCTAGCTATCGCCATGTAAGCCCACTGCAA
GCTACGCCCGGGCCTTGTACAGCTCGTCCATGCCGAGAGTGATCCCGGCGGCGGTCACGAACTCCAGCAGGACCATGTGATCGCGCTTCTCGTTGGGGTC
TTTGCTCAGGGCGGACTGGGTGCTCAGGTAGTGGTTGTCGGGCAGCAGCACGGGGCCGTCGCCGATGGGGGTGTTCTGCTGGTAGTGGTCGGCGAGCTGC
ACGCTGCCGTCCTCGATGTTGTGGCGGATCTTGAAGTTCACCTTGATGCCGTTCTTCTGCTTGTCGGCCATGATATAGACGTTGTGGCTGTTGTAGTTGT
ACTCCAGCTTGTGCCCCAGGATGTTGCCGTCCTCCTTGAAGTCGATGCCCTTCAGCTCGATGCGGTTCACCAGGGTGTCGCCCTCGAACTTCACCTCGGC
GCGGGTCTTGTAGTTGCCGTCGTCCTTGAAGAAGATGGTGCGCTCCTGGACGTAGCCTTCGGGCATGGCGGACTTGAAGAAGTCGTGCTGCTTCATGTGG
TCGGGGTAGCGGCTGAAGCACTGCACGCCGTAGGTCAGGGTGGTCACGAGGGTGGGCCAGGGCACGGGCAGCTTGCCGGTGGTGCAGATGAACTTCAGGG
TCAGCTTGCCGTAGGTGGCATCGCCCTCGCCCTCGCCGGACACGCTGAACTTGTGGCCGTTTACGTCGCCGTCCAGCTCGACCAGGATGGGCACCACCCC
GGTGAACAGCTCCTCGCCCTTGCTGGCGCGCCAGATGTGTATAAGAGACAGAAGGGCGAATTCTGCAGATATCCATCACACTGGCGGCCGCTCGAGCATG
CATCTAGAGGGCCCAATTCGCCCTATAGTGAGTCGTATTACAATTCACTGGCCGTCGTTTTACAACGTCGTGACTGGGAAAACCCTGGCGTTACCCAACT
TAATCGCCTTGCAGCACATCCCCCTTTCGCCAGCTGGCGTAATAGCGAAGAGGCCCGCACCGATCGCCCTTCCCAACAGTTGCGCAGCCTATACGTACGG
CAGTTTAAGGTTTACACCTATAAAAGAGAGAGCCGTTATCGTCTGTTTGTGGATGTACAGAGTGATATTATTGACACGCCGGGGCGACGGATGGTGATCC
CCCTGGCCAGTGCACGTCTGCTGTCAGATAAAGTCTCCCGTGAACTTTACCCGGTGGTGCATATCGGGGATGAAAGCTGGCGCATGATGACCACCGATAT
GGCCAGTGTGCCGGTCTCCGTTATCGGGGAAGAAGTGGCTGATCTCAGCCACCGCGAAAATGACATCAAAAACGCCATTAACCTGATGTTCTGGGGAATA
TAAATGTCAGGCATGAGATTATCAAAAAGGATCTTCACCTAGATCCTTTTCACGTAGAAAGCCAGTCCGCAGAAACGGTGCTGACCCCGGATGAATGTCA
GCTACTGGGCTATCTGGACAAGGGAAAACGCAAGCGCAAAGAGAAAGCAGGTAGCTTGCAGTGGGCTTACATGGCGATAGCTAGACTGGGCGGTTTTATG
GACAGCAAGCGAACCGGAATTGCCAGCTGGGGCGCCCTCTGGTAAGGTTGGGAAGCCCTGCAAAGTAAACTGGATGGCTTTCTCGCCGCCAAGGATCTGA
TGGCGCAGGGGATCAAGCTCTGATCAAGAGACAGGATGAGGATCGTTTCGCATGATTGAACAAGATGGATTGCACGCAGGTTCTCCGGCCGCTTGGGTGG
AGAGGCTATTCGGCTATGACTGGGCACAACAGACAATCGGCTGCTCTGATGCCGCCGTGTTCCGGCTGTCAGCGCAGGGGCGCCCGGTTCTTTTTGTCAA
GACCGACCTGTCCGGTGCCCTGAATGAACTGCAAGACGAGGCAGCGCGGCTATCGTGGCTGGCCACGACGGGCGTTCCTTGCGCAGCTGTGCTCGACGTT
GTCACTGAAGCGGGAAGGGACTGGCTGCTATTGGGCGAAGTGCCGGGGCAGGATCTCCTGTCATCTCACCTTGCTCCTGCCGAGAAAGTATCCATCATGG
CTGATGCAATGCGGCGGCTGCATACGCTTGATCCGGCTACCTGCCCATTCGACCACCAAGCGAAACATCGCATCGAGCGAGCACGTACTCGGATGGAAGC
CGGTCTTGTCGATCAGGATGATCTGGACGAAGAGCATCAGGGGCTCGCGCCAGCCGAACTGTTCGCCAGGCTCAAGGCGAGCATGCCCGACGGCGAGGAT
CTCGTCGTGACCCATGGCGATGCCTGCTTGCCGAATATCATGGTGGAAAATGGCCGCTTTTCTGGATTCATCGACTGTGGCCGGCTGGGTGTGGCGGACC
GCTATCAGGACATAGCGTTGGCTACCCGTGATATTGCTGAAGAGCTTGGCGGCGAATGGGCTGACCGCTTCCTCGTGCTTTACGGTATCGCCGCTCCCGA
TTCGCAGCGCATCGCCTTCTATCGCCTTCTTGACGAGTTCTTCTGAATTATTAACGCTTACAATTTCCTGATGCGGTATTTTCTCCTTACGCATCTGTGC
GGTATTTCACACCGCATACAGGTGGCACTTTTCGGGGAAATGTGCGCGGAACCCCTATTTGTTTATTTTTCTAAATACATTCAAATATGTATCCGCTCAT
GAGACAATAACCCTGATAAATGCTTCAATAATAGCACGTGAGGAGGGCCACCATGGCCAAGTTGACCAGTGCCGTTCCGGTGCTCACCGCGCGCGACGTC
GCCGGAGCGGTCGAGTTCTGGACCGACCGGCTCGGGTTCTCCCGGGACTTCGTGGAGGACGACTTCGCCGGTGTGGTCCGGGACGACGTGACCCTGTTCA
TCAGCGCGGTCCAGGACCAGGTGGTGCCGGACAACACCCTGGCCTGGGTGTGGGTGCGCGGCCTGGACGAGCTGTACGCCGAGTGGTCGGAGGTCGTGTC
CACGAACTTCCGGGACGCCTCCGGGCCGGCCATGACCGAGATCGGCGAGCAGCCGTGGGGGCGGGAGTTCGCCCTGCGCGACCCGGCCGGCAACTGCGTG
CACTTCGTGGCCGAGGAGCAGGACTGACACGTGCTAAAACTTCATTTTTAATTTAAAAGGATCTAGGTGAAGATCCTTTTTGATAATCTCATGACCAAAA
TCCCTTAACGTGAGTTTTCGTTCCACTGAGCGTCAGACCCCGTAGAAAAGATCAAAGGATCTTCTTGAGATCCTTTTTTTCTGCGCGTAATCTGCTGCTT
GCAAACAAAAAAACCACCGCTACCAGCGGTGGTTTGTTTGCCGGATCAAGAGCTACCAACTCTTTTTCCGAAGGTAACTGGCTTCAGCAGAGCGCAGATA
CCAAATACTGTCCTTCTAGTGTAGCCGTAGTTAGGCCACCACTTCAAGAACTCTGTAGCACCGCCTACATACCTCGCTCTGCTAATCCTGTTACCAGTGG
CTGCTGCCAGTGGCGATAAGTCGTGTCTTACCGGGTTGGACTCAAGACGATAGTTACCGGATAAGGCGCAGCGGTCGGGCTGAACGGGGGGTTCGTGCAC
ACAGCCCAGCTTGGAGCGAACGACCTACACCGAACTGAGATACCTACAGCGTGAGCTATGAGAAAGCGCCACGCTTCCCGAAGGGAGAAAGGCGGACAGG
TATCCGGTAAGCGGCAGGGTCGGAACAGGAGAGCGCACGAGGGAGCTTCCAGGGGGAAACGCCTGGTATCTTTATAGTCCTGTCGGGTTTCGCCACCTCT
GACTTGAGCGTCGATTTTTGTGATGCTCGTCAGGGGGGCGGAGCCTATGGAAAAACGCCAGCAACGCGGCCTTTTTACGGTTCCTGGCCTTTTGCTGGCC
TTTTGCTCACATGTTCTTTCCTGCGTTATCCCCTGATTCTGTGGATAACCGTATTACCGCCTTTGAGTGAGCTGATACCGCTCGCCGCAGCCGAACGACC
GAGCGCAGCGAGTCAGTGAGCGAGGAAGCGGAAG

pBNJ12.5

Prestin cDNA in an Amp resisant CMV expression plasmid (7029 bp)

CMV promoter	488-1013
SP6 promoter	1111-1128
Prestin ORF	1408-3639
SV40 poly A	4200-4428
f1 (-) ori	4834-4566
f1 (+) ori	4860-5011
Amp resistance ORF	5309-6166
pMB1 ori	6356-7029

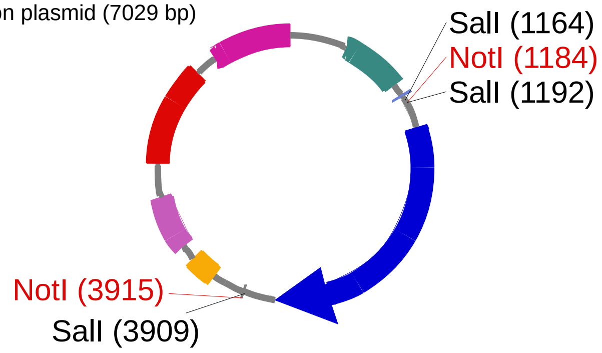


```
GGCCGCTCTAGCTTGGGATCTTTGTGAAGGAACCTTACTTCTGTGGTGTGACATAATTGGACAAACTACCTACAGAGATTTAAAGCTCTAAGGTAAATATA
AAATTTTTAAGTGTATAATGTGTTAAACTAGCTGCATATGCTTGCTGCTTGAGAGTTTTGCTTACTGAGTATGATTTATGAAAATATTATACACAGGAGCT
AGTGATTCTAATTGTTTGTGTATTTTAGATTCACAGTCCCAAGGCTCATTTCAGGCCCCTCAGTCCTCACAGTCTGTTCATGATCATAATCAGCCATACCA
CATTTGTAGAGGTTTTACTTGCTTTAAAAAACCTCCCACACCTCCCCCTGAACCTGAAACATAAAATGAATGCAATTGTTGTTGTTAACTTGTTTATTGCA
GCTTATAATGGTTACAAATAAAGCAATAGCATCACAAATTTCACAAATAAAGCATTTTTTTCACTGCATTCTAGTTGTGGTTTGTCCAAACTCATCAATGT
ATCTTATCATGTCTGGATCGATCCTGCATTAATGAATCGGCCAACGCGCGGGGAGAGGCGGTTTGCGTATTGGCTGGCGTAATAGCGAAGAGGCCCGCACC
GATCGCCCTTCCCAACAGTTGCGCAGCCTGAATGGCGAATGGACGCGCCCTGTAGCGGCGCATTAAGCGCGGCGGGTGTGGTGGTTACGCGCAGCGTGACC
GCTACACTTGCCAGCGCCCTAGCGCCCGCTCCTTTCGCTTTCTTCCCTTCCTTTCTCGCCACGTTCGCCGGCTTTCCCCGTCAAGCTCTAAATCGGGGGCT
CCCTTTAGGGTTCCGATTTAGTGCTTTACGGCACCTCGACCCCAAAAAACTTGATTAGGGTGATGGTTCACGTAGTGGGCCATCGCCCTGATAGACGGTTT
TTCGCCCTTTGACGTTGGAGTCCACGTTCTTTAATAGTGGACTCTTGTTCCAAACTGGAACAACACTCAACCCTATCTCGGTCTATTCTTTTGATTTATAA
GGGATTTTGCCGATTTCGGCCTATTGGTTAAAAAATGAGCTGATTTAACAAAAATTTAACGCGAATTTTAACAAAATATTAACGTTTACAATTTCAGGTGG
CACTTTTCGGGGAAATGTGCGCGGAACCCCTATTTGTTTATTTTTCTAAATACATTCAAATATGTATCCGCTCATGCCAGGTCTTGGACTGGTGAGAACGG
CTTGCTCGGCAGCTTCGATGTGTGCTGGAGGGAGAATAAAGGTCTAAGATGTGCGATAGAGGGAAGTCGCATTGAATTATGTGCTGTGTAGGGATCGCTGG
TATCAAATATGTGTGCCCACCCCTGGCATGAGACAATAACCCTGATAAATGCTTCAATAATATTGAAAAAGGAAGAGTATGAGTATTCAACATTTCCGTGT
CGCCCTTATTCCCTTTTTTGCGGCATTTTGCCTTCCTGTTTTTGCTCACCCAGAAACGCTGGTGAAAGTAAAAGATGCTGAAGATCAGTTGGGTGCACGAG
TGGGTTACATCGAACTGGATCTCAACAGCGGTAAGATCCTTGAGAGTTTTCGCCCCGAAGAACGTTTTCCAATGATGAGCACTTTTAAAGTTCTGCTATGT
GGCGCGGTATTATCCCGTATTGACGCCGGGCAAGAGCAACTCGGTCGCCGCATACACTATTCTCAGAATGACTTGGTTGAGTACTCACCAGTCACAGAAAA
GCATCTTACGGATGGCATGACAGTAAGAGAATTATGCAGTGCTGCCATAACCATGAGTGATAACACTGCGGCCAACTTACTTCTGACAACGATCGGAGGAC
CGAAGGAGCTAACCGCTTTTTTGCACAACATGGGGGATCATGTAACTCGCCTTGATCGTTGGGAACCGGAGCTGAATGAAGCCATACCAAACGACGAGCGT
GACACCACGATGCCTGTAGCAATGGCAACAACGTTGCGCAAACTATTAACTGGCGAACTACTTACTCTAGCTTCCCGGCAACAATTAATAGACTGGATGGA
GGCGGATAAAGTTGCAGGACCACTTCTGCGCTCGGCCCTTCCGGCTGGCTGGTTTATTGCTGATAAATCTGGAGCCGGTGAGCGTGGGTCTCGCGGTATCA
TTGCAGCACTGGGGCCAGATGGTAAGCCCTCCCGTATCGTAGTTATCTACACGACGGGGAGTCAGGCAACTATGGATGAACGAAATAGACAGATCGCTGAG
ATAGGTGCCTCACTGATTAAGCATTGGTAACTGTCAGACCAAGTTTACTCATATATACTTTAGATTGATTTAAAACTTCATTTTTAATTTAAAAGGATCTA
GGTGAAGATCCTTTTTGATAATCTCATGCCATAACTTCGTATAATGTATGCTATACGAAGTTATGGCATGACCAAAATCCCTTAACGTGAGTTTTCGTTCC
ACTGAGCGTCAGACCCCGTAGAAAAGATCAAAGGATCTTCTTGAGATCCTTTTTTTCTGCGCGTAATCTGCTGCTTGCAAACAAAAAAACCACCGCTACCA
GCGGTGGTTTGTTTGCCGGATCAAGAGCTACCAACTCTTTTTCCGAAGGTAACTGGCTTCAGCAGAGCGCAGATACCAAATACTGTCCTTCTAGTGTAGCC
GTAGTTAGGCCACCACTTCAAGAACTCTGTAGCACCGCCTACATACCTCGCTCTGCTAATCCTGTTACCAGTGGCTGCTGCCAGTGGCGATAAGTCGTGTC
TTACCGGGTTGGACTCAAGACGATAGTTACCGGATAAGGCGCAGCGGTCGGGCTGAACGGGGGGTTCGTGCACACAGCCCAGCTTGGAGCGAACGACCTAC
ACCGAACTGAGATACCTACAGCGTGAGCATTGAGAAAGCGCCACGCTTCCCGAAGGGAGAAAGGCGGACAGGTATCCGGTAAGCGGCAGGGTCGGAACAGG
AGAGCGCACGAGGGAGCTTCCAGGGGGAAACGCCTGGTATCTTTATAGTCCTGTCGGGTTTCGCCACCTCTGACTTGAGCGTCGATTTTTGTGATGCTCGT
CAGGGGGGCGGAGCCTATGGAAAAACGCCAGCAACGCGGCCTTTTTACGGTTCCTGGCCTTTTGCTGGCCTTTTGCTCACATGTTCTTTCCTGCGTTATCC
CCTGATTCTGTGGATAACCGTATTACCGCCTTTGAGTGAGCTGATACCGCTCGCCGCAGCCGAACGACCGAGCGCAGCGAGTCAGTGAGCGAGGAAGCGGA
AGAGCGCCCAATACGCAAACCGCCTCTCCCCGCGCGTTGGCCGATTCATTAATGCAGAGCTTGCAATTCGCGCGTTTTTCAATATTATTGAAGCATTTATC
AGGGTTATTGTCTCATGAGCGGATACATATTTGAATGTATTTAGAAAAATAAACAAATAGGGGTTCCGCGCACATTTCCCCGAAAAGTGCCACCTGACGTC
TAAGAAACCATTATTATCATGACATTAACCTATAAAAATAGGCGTATACGAGGCCCTTTCACTCATTAGGCACCCCAGGCTTTACACTTTATAGCTTCCGG
CTCGTATAATGTGTGGAATTGTGAGCGGATAACAATTTCACACAGGAAACAGCATCGTGCAGGTCGTTACATAACTTACGGTAAATGGCCCGCCTGGCTGA
CCGCCCAACGACCCCCGCCCATTGACGTCAATAATGACGTATGTTCCCATAGTAACGCCAATAGGGACTTTCCATTGACGTCAATGGGTGGAGTATTTACG
GTAAACTGCCCACTTGGCAGTACATCAAGTGTATCATATGCCAAGTACGCCCCCTATTGACGTCAATGACGGTAAATGGCCCGCCTGGCATTATGCCCAGT
ACATGACCTTATGGGACTTTCCTACTTGGCAGTACATCTACGTATTAGTCATCGCTATTACCATGGTGATGCGGTTTTGGCAGTACATCAATGGGCGTGGA
TAGCGGTTTGACTCACGGGGATTTCCAAGTCTCCACCCCATTGACGTCAATGGGAGTTTGTTTTGGCACCAAAATCAACGGGACTTTCCAAAATGTCGTAA
CAACTCCGCCCCATTGACGCAAATGGGCGGTAGGCGTGTACGGTGGGAGGTCTATATAAGCAGAGCTCGTTTAGTGAACCGTCAGATCGCCTGGAGACGCC
ATCCACGCTGTTTTGACCTCCATAGAAGACACCGGGACCGATCCAGCCTCCGGACTCTAGCCTAGGCTTTTGCAAAAAGCTATTTAGGTGACACTATAGAA
GGTACGCCTGCAGGTACCGGTCCGGAATTCCCGGGTCGACGAGCTCACTAGTCGGCGGCCGCGTCGACGGCAGCGGCGGCTCCGCCCTGCGCAGCCCCGGC
AGCGCTGGCTGGTGGCGGGGGAGGGCGAACAGTCCCTTTTCCAGCCCTCAGCAGTTGACTGCCCTGTACCTGGAGTTCCCAGCCGGCTTTCCATCCCCTGT
ACCTTGTGCTATACGTTGGATCTGGTGCCTGTCCAGAAATGCTCGTCTCCTGCTGTTGGTGAATAACTGCAGACCATGGATCATGCCGAAGAAAATGAAAT
CCCTGTGGCAACCCAGAAGTACCACGTGGAAAGGCCTATCTTCAGTCATCCCGTCCTCCAGGAGAGGCTGCATGTCAAGGACAAAGTCTCAGAGTCCATTG
GGGATAAGCTGAAGCAGGCGTTCACATGCACTCCCAAAAAGATAAGAAACATCATTTACATGTTCCTGCCCATCACTAAGTGGTTGCCAGCTTACAAGTTC
AAGGAGTATGTGTTGGGTGACTTGGTTTCAGGCATAAGCACCGGCGTGCTTCAGCTTCCCCAAGGCTTAGCCTTCGCAATGCTCGCGGCTGTTCCTCCGGT
GTTCGGCCTGTACTCTTCATTTTATCCTGTTATCATGTACTGTTTCTTTGGGACCTCCAGACACATATCTATAGGTCCTTTCGCCGTCATTAGTTTGATGA
TCGGTGGTGTGGCTGTCCGGCTGGTCCCCGATGACATCGTCATCCCGGGAGGAGTGAACGCAACCAACGGCACGGAGGCCCGAGACGCGCTGAGAGTGAAA
GTCGCCATGTCTGTCACCCTGCTCTCAGGAATCATTCAGTTTTGCCTAGGTGTGTGCAGGTTTGGATTTGTGGCCATATACCTCACGGAGCCGCTGGTGCG
AGGGTTCACCACCGCCGCCGCCGTGCACGTCTTCACATCCATGTTGAAATACCTGTTTGGGGTTAAGACAAAGCGGTACAGTGGGATCTTTTCGGTGGTAT
ATAGTACAGTTGCTGTGTTGCAGAATGTTAAAAACCTCAACGTGTGTTCCCTAGGCGTCGGCCTGATGGTTTTTGGTTTGCTGTTGGGTGGCAAGGAGTTT
AATGAGAGATTTAAAGAGAAATTGCCAGCACCCATTCCTCTAGAGTTCTTTGCTGTGGTGATGGGAACTGGCATTTCCGCGGGGTTTAACTTGCACGAGTC
CTACAGTGTGGATGTCGTTGGAACTCTTCCTCTGGGGCTACTCCCTCCTGCCAACCCGGACACCAGCCTCTTCCACCTCGTGTATGTGGATGCCATTGCCA
TAGCCATCGTTGGATTTTCAGTGACAATTTCCATGGCCAAAACCTTGGCGAATAAGCATGGCTACCAGGTTGATGGCAATCAGGAGCTCATCGCTTTGGGG
ATATGCAACTCCATCGGATCTCTCTTCCAGACCTTCTCCATTTCCTGCTCCTTGTCTCGCAGCCTTGTTCAGGAGGGAACTGGAGGGAAAACACAGCTCGC
AGGTTGCTTGGCCTCGCTGATGATTCTGCTGGTCATTTTAGCCACTGGATTCCTCTTTGAGTCATTGCCCCAGGCTGTGCTCTCGGCCATTGTGATCGTGA
ACCTGAAAGGGATGTTTATGCAGTTCTCAGATCTGCCCTTCTTCTGGAGAACCAGCAAAATAGAGCTGACCATCTGGCTTACCACCTTTGTGTCCTCCCTG
TTCCTGGGCTTGGACTACGGACTGATTACTGCTGTGATCATTGCTCTGCTGACTGTGATTTACAGAACCCAGAGTCCGAGCTACAAGGTCCTGGGGCAGCT
CCCTGACACCGATGTATACATTGACATAGACGCATATGAGGAGGTGAAAGAAATTCCTGGAATAAAAATATTCCAGATAAACGCCCCAATTTACTATGCAA
ACAGTGACTTGTATAGCAACGCCCTAAAAAGAAAGACTGGTGTGAACCCAGCGCTCATAATGGGAGCAAGAAGGAAGGCCATGAGGAAGTACGCAAAGGAA
GTCGGAAACGCCAACATTGCCAACGCAGCTGTTGTCAAAGTGGATGGAGAAGTAGATGGAGAAAATGCTACGAAGCCCGAAGAAGAGGATGATGAAGTAAA
ATATCCCCCAATAGTCATCAAAACAACATTTCCTGAAGAGCTGCAGAGATTTATGCCCCAGACAGAAAATGTCCACACTATCATTCTAGACTTCACACAAG
TCAATTTTATCGACTCTGTTGGAGTAAAAACCCTGGCTGTGATGGTGAAGGAATACGGAGATGTTGGTATTTATGTGTACTTAGCAGGATGCAGCCCACAA
GTCGTGAATGACCTCACCCGCAACCGTTTCTTTGAAAATCCTGCCTTAAAAGAGCTTCTGTTCCACAGTATCCATGATGCAGTCTTGGGCAGCCATGTTCG
AGAGGCAATGGCTGAGCAAGAAGCCTCAGCCCCACCTCCCCAGGACGACATGGAGCCCAATGCCACACCCACCACACCCGAGGCATAAAGGGCCTGCCTGG
GCCTGTGCACCTCTTGAATTCTGAACTTACATGCTTTAAATACCAGGCCTTAGGTTTTCTTCTACCCAACCCCCAACCCCCAAGGAAAATGTTAGTAGTTA
TGGCTTGATTTGGAGGGTGAATGATGTGTAGTGCGATGTATCTCAGACTTGTGTTATTTTATCTGAATAATTCAAAGATTAAGTGGCCTTTCGCACTTATG
TAGTGATGTTTGTATTATATCTTAAAGTCAAAAAAAAAAAAAAAAAAAAAAAAGTCGACGC
```
